# Targeting stemness pathways modulates macrophage polarization and reprograms the tumor microenvironment

**DOI:** 10.3389/fimmu.2025.1513404

**Published:** 2025-03-14

**Authors:** Austeja Butkute, Marius Baltramonaitis, Simona Malmige, Adas Darinskas, Vita Pasukoniene, Agata Mlynska

**Affiliations:** ^1^ Laboratory of Immunology, National Cancer Institute, Vilnius, Lithuania; ^2^ Life Sciences Center, Vilnius University, Vilnius, Lithuania; ^3^ STEAM Center, Vilnius University, Vilnius, Lithuania; ^4^ Department of Chemistry and Bioengineering, Vilnius Gediminas Technical University, Vilnius, Lithuania

**Keywords:** colorectal cancer, breast cancer, tumor microenvironment, macrophage polarization, M1/M2 phenotype, stemness inhibition, combination therapy

## Abstract

**Introduction:**

The tumor microenvironment plays a pivotal role in cancer progression and therapeutic resistance, with tumor-associated macrophages significantly influencing immune suppression and tumor growth. Colorectal cancers (CRC) classified as Consensus Molecular Subtype 4 (CMS4) and triple-negative breast cancers subsets are particularly characterized by a mesenchymal phenotype, immune exclusion, and extensive macrophage infiltration. This study aimed to investigate how targeting cancer cell stemness with specific inhibitors could modulate macrophage polarization in CRC *in vitro* and breast cancer *in vivo*, potentially shifting the immune balance from pro-tumor M2-like to anti-tumor M1-like macrophages.

**Methods:**

We used four stemness inhibitors—salinomycin, SB-431542, JIB-04, and napabucasin—each targeting different pathways (Wnt/β-catenin, TGF-β, histone demethylation, and STAT3, respectively), to evaluate their effects on CMS4 CRC cell lines (HCT116 and SW620) and human peripheral blood-derived macrophages in an indirect co-culture model.

**Results:**

Our results showed that CMS4 CRC cell lines induced distinct macrophage polarization patterns, with HCT116 promoting M2-like macrophages and SW620 leaning toward M1-like profile. Notably, the combination of stemness inhibitors reduced stemness markers (CD133, CD44) in colorectal cancer cells and shifted macrophage polarization toward an M1-like phenotype, particularly in co-culture with HCT116. *In vivo* studies using the syngeneic immunocompetent EO771 breast cancer mouse model demonstrated that combination of stemness inhibitors increased the M1/M2 macrophage ratio.

**Conclusions:**

Our study highlights the dual potential of stemness inhibitors to target both cancer cells and the immune microenvironment. These findings offer promising strategies for enhancing favorable immunomodulation in mesenchymal-like colorectal tumors.

## 1 Introduction

The tumor microenvironment (TME) plays a crucial role in cancer progression and therapeutic resistance. Composed of a complex mix of stromal cells, immune cells, and extracellular matrix components, the TME interacts with cancer cells to create a supportive niche for tumor growth and immune evasion (1). Cancer therapies targeting the interactions in the TME have gained significant attention in recent years. Given the heterogeneity of solid tumors and the substantial proportion of non-cancer cells within the tumor mass, it is crucial to assess the effects of novel anticancer therapies not only on cancer cells but also on the surrounding TME. Within the TME, macrophages are among the most abundant immune cells, making up approximately 50 % of tumor-infiltrating immune cells (2). These tumor-associated macrophages are highly plastic and can exhibit a spectrum of activation states between the pro-inflammatory, anti-tumor M1 phenotype and the anti-inflammatory, pro-tumor M2 phenotype. Macrophage polarization is influenced by signals from tumor cells. Tumor-associated macrophages can drive tumor growth, angiogenesis, metastasis, and immune regulation (3), particularly through immunosuppressive signaling (4) and the promotion of effector T cell immune exclusion (5).

Colorectal cancer (CRC) and breast cancer are among the most frequently diagnosed solid tumors (6), both characterized by stroma-rich microenvironments (7) with substantial macrophage infiltration, which is associated with distinct clinical outcomes (8). CRC, a highly heterogeneous disease, has been classified into four Consensus Molecular Subtypes (CMS) based on gene expression profiles (9). These subtypes reflect distinct biological and clinical characteristics. CMS1 tumors, for example, are immune-active, associated with high immune infiltration and favorable prognosis, whereas CMS4 tumors are mesenchymal in nature and are marked by poor prognosis, high stromal content, and extensive macrophage infiltration Additionally, CMS4 tumors often display features of immune exclusion (10), where T cells, despite being present in the TME, are unable to effectively penetrate or act against tumor cells (11). CMS4 subtype tumors are infiltrated with M2 macrophages, monocytes, resting DCs, Tregs and eosinophils, but levels of activated DCs, NK cells, and M1-like macrophages are low (12). Importantly, M2-like macrophages impair the target recognition function of CD4+ T cells and tumor-killing functions of CD8+ T cells (13). Various signaling pathways, including TGF-β, Hedgehog, WNT/β-catenin, and other, are active in CMS4 CRC cells, driving the maintenance of stemness properties and immunosuppression through signaling molecules (14). This stromal-rich, immune-suppressive environment complicates therapeutic interventions, as effector immune cells are often physically and functionally hindered from targeting cancer cells.

Breast cancer is a diverse and multifaceted disease strongly associated with immunomodulatory processes, orchestrated by tumor-associated macrophages, T cells, natural killer cells, and B cells, among others (15). Immune escape in breast cancer can be mediated via the PD-1/PD-L1 pathway (16), which was shown to be activated by M2 macrophages and TGF-β signaling (17). M2-like macrophages also secrete substantia amounts of VEGF (18), often limiting T cell trafficking into tumors. The immune landscape of breast cancer is complex and diverse, displaying considerable differences between patients, subtypes, and disease stages. Tumor-resident macrophages contribute to early development, recurrence and metastasis of triple-negative breast cancer (TNBC) subset (19), associated with worst survival among all breast cancer subtypes (20).

Stemness properties in cancer cells play a crucial role in modulating the TME, especially through interactions of cancer cells with tumor-associated macrophages and particularly in mesenchymal-like tumors such as CMS4 colorectal cancers (21) and triple-negative breast cancer (22). Increased stemness properties in cancer cells are associated with a reduction in anti-tumor immune cells, including CD8+ cells, natural killer (NK) and B cells within the TME, and elevated proportion of tumor-associated macrophages (18). Targeting stemness in cancer cells, therefore, not only impacts the tumor directly but also holds potential for reprogramming the TME by influencing macrophage plasticity. By modulating key pathways associated with cancer cell stemness—such as Wnt/β-catenin, Notch, Hedgehos, TGF-β, PI3K/Akt or STAT—it may be possible to indirectly affect macrophage polarization a tumor-promoting to a tumor-suppressive state (23, 24). However, despite the known efficacy of some stemness inhibitors in reducing stemness properties in cancer cells, their direct off-target impact on tumor-associated macrophages and the immune TME remains underexplored, highlighting the need for further research to understand the dynamics and develop more effective therapies.

Despite considerable advancements in understanding the macrophage biology across CRC and breast cancer subtypes, a key gap persists in how stemness-targeting therapies influence the immune TME, particularly in mesenchymal-rich tumors like CMS4 CRC or TNBC. In this study, we hypothesized that different stemness inhibitors, whether used individually or in combination, could reprogram macrophages from a tumor-promoting M2-like phenotype to an anti-tumor M1-like phenotype, thereby altering the TME in favor of anti-tumor immunity. Our objectives were to explore the macrophage polarization patterns elicited by CRC cell lines representing various CMS subtypes and to assess the effects of stemness inhibitors on both CRC cells and macrophages in an indirect co-culture model *in vitro*. Furthermore, we aimed to investigate whether these findings could be translated into an *in vivo* TNBC context, providing insights into the therapeutic potential of targeting stemness in the broader tumor microenvironment.

## 2 Materials and methods

### 2.1 Cancer cell lines

Human colorectal cancer cell lines LoVo, HCT 116, SW620, HT-29, DLD1, LS1034, NCI-H508, COLO320, and mouse breast cancer cell lines EO771 and 4T1 were obtained from American Type Culture Collection (ATCC). The cell lines were grown in RPMI-1640 (Gibco, cat. no. 61870-010) supplemented with 10 % FBS (Gibco, cat. no. A5256801) and 1 % of antibiotics (100 U/mL penicillin and 100 μg/mL streptomycin, P/S) (Gibco, cat. no. 15140-122). During all experiments cells were maintained at 37°C in a humidified atmosphere at 5 % CO_2_ and all cell lines regularly passaged after reaching confluence. For *in vitro* experiments, human colorectal cancer cell lines were used at passages 15-30, while for *in vivo* experiments, mouse breast cancer cell lines were used at passages 4-6.

### 2.2 Animals

C57BL/6 mice were originally purchased from Inotiv and bred in-house for 8 months prior to experimentation. Animals were housed in standard conditions, with 5-7 mice per cage, which were cleaned weekly. The mice were kept in an animal facility with controlled environmental conditions, including a temperature of 22-24°C, humidity levels of 40-60%, and a 12-hour light/dark cycle. They had free access to food (chow diet) and water.

### 2.3 Drugs

Four stemness inhibitors were used for this study: salinomycin (Sigma-Aldrich, cat no. S4526) for targeting Wnt/β-catenin pathway; SB-431542 (Tocris Bioscience, cat. no. 301836-41-9) for targeting TGF-β; JIB-04 (Tocris Bioscience, cat. no. 199596-05-9) for histone demethylation; napabucasin (Tocris Bioscience, cat.no. 83280-65-3) for targeting STAT3. Stock solutions were prepared in DMSO (Carl Roth, cat.no A994.2 ) at a concentration of 50 mg/mL (salinomycin, SB-431542, JIB-04) or 20 mg/ml (napabucasin) and diluted in RPMI medium for use in assays.

### 2.4 Macrophage differentiation from peripheral blood mononuclear cells

Peripheral blood mononuclear cells (PBMCs) were isolated individually from the leukocyte- and platelet-rich fraction (L-PRF) obtained from five different donors. Each donor’s L-PRF sample was processed independently and there were no pooling of samples throughout the study. To isolate PBMCs, L-PRF was layered onto Ficoll-Paque (Cytiva, cat. no. 17144003) at a ratio 2:1 and centrifuged at 1000 g for 30 minutes without brake. The PBMC layer was aspirated and washed three times with phosphate-buffered saline (PBS; Gibco, cat. no. 10010-015) at 500 g for 7 minutes. The PBMCs were then counted and seeded at 3 × 10^6 cells in 10 ml of serum-free RPMI medium in a 100 mm diameter Petri dish and allowed to adhere for 24 hours.

The following day, the medium containing non-adherent lymphocytes was aspirated, and cells were washed with PBS. Adherent monocytes were detached using Trypsin/EDTA solution (Gibco, cat. no. 25200-056), collected, counted using an Adam cell counter (NanoEntek), and evaluated by flow cytometry (LSR II, Becton Dickinson) to assess the proportion of monocytes in the population. Monocytes were then seeded in six-well plates at a density of 5 × 10^5 cells per well in 2 ml of RPMI medium supplemented with 10% serum.

To differentiate into macrophages, monocytes were cultured with 20 ng/ml M-CSF (BioLegend, cat. no. 574804) for six days, with a medium change on day three. For polarization controls, M0 macrophages were polarized to the M1-like type using 15 ng/ml lipopolysaccharide (LPS; Merck Millipore, cat. no. LPS25) and 25 ng/ml IFNγ (BioLegend, cat.no. 570206), or to the M2-like type using 25 ng/ml IL-4 (BioLegend, cat. no. 574004), for an additional 48 hours.

### 2.5 Indirect colorectal cancer cell and macrophage co-culture

Twenty-four hours prior to initiating co-culture, 2 × 10^5 colorectal cancer (CRC) cells were seeded into six-well plates containing 0.4 μm pore transparent PET transwell inserts (Sarstedt) in supplemented RPMI medium. The following day, the transwell inserts containing the CRC cells were transferred into another six-well plate containing differentiated macrophages (day 6 post-seeding). All media were refreshed with supplemented RPMI medium and cells were co-cultured for 48 hours. Co-cultures were performed in technical duplicates across four independent repeats, followed by further phenotypic analysis.

### 2.6 Flow cytometry

Cell viability and changes in surface marker expression were assessed using flow cytometry. Cells were detached from the growth surface and transferred to a cytometric tube. The cell suspension was washed with 200 µL of PBS and centrifuged at 300 g for 5 minutes. After centrifugation, the supernatant was discarded, and fluorochrome-labeled antibodies were added. The list of staining panels including antibodies used, their fluorochrome conjugates, and suppliers are provided in **Table 1**. Single-stain controls were prepared for compensation. Fluorescence-minus-one controls were used in multicolor panels to set gating thresholds for each marker. The samples were incubated with antibodies for 20 minutes in the dark at 4°C. Following incubation, unbound antibodies were removed by washing with 200 µL of PBS, followed by centrifugation at 300 g for 5 minutes. The cells were then resuspended in 200 µL of PBS for flow cytometry analysis.

**Table 1:** **List of staining panels for flow cytometry**.

Target	Label	Clone	Manufacturer	Cat. No.	Dilution	Markers
Panel 1A – human macrophage phenotyping						
CD86	FITC	BU63	EXBIO	1F-531-T100	1:25	M1 macrophage
CD80	V450	16-10A1	BD Biosciences	560442	1:100	M1 macrophage
CD11c	PE	B-ly6	BD Bioscience	555392	1:100	M1 macrophage
Panel 1B – human macrophage phenotyping						
MHC II	APC	L243	BD Biosciences	347403	1:100	M1 macrophage
CD274	FITC	29E.2A3	BD Biosciences	558065	1:25	M1 macrophage
CD163	PE	RM3/1	BD Biosciences	567881	1:50	M2 macrophage
CD206	BV421	15-2	BioLegend	321125	1:100	M2 macrophage
Panel 2A – human cancer cell phenotyping						
CD133	PE	S16016B	Miltenyi Biotec	130-090-853	1:100	Stemness
CD44	APC	BJ18	Miltenyi Biotec	130-095-177	1:100	Stemness
Panel 2B – human cancer cell phenotyping						
ESA	APC	9C4	BD Biosciences	566842	1:100	Stemness
MHC I	FITC	BB7.2	BD Biosciences	557348	1:25	Immunomodulation
CD274	PE	29E.2A3	BD Biosciences	557924	1:50	Immunomodulation
Panel 3 – mouse cancer cell phenotyping						
CD44	APC	C068C2	BioLegend	397506	1:400	Stemness
CD274	PE-Cy7	10F.9G2	BioLegend	329718	1:100	Immunomodulation

Detailed information regarding antibody target, label, clone, manufacturer and catalog number, dilution, and function is provided.

Flow cytometric analysis was performed using a LSR II flow cytometer (Becton Dickinson), and data were processed with FACSDiva software. A minimum of 20,000 events per sample were recorded. Cells morphology was analyzed for size (forward scatter) and granularity (side scatter), gating on live cells to exclude debris and doublets. Cell phenotype was analyzed on gated cells by extracting the median fluorescence intensity signal for each marker individually.

For presentation of flow cytometry data in heatmaps, modified z-score or log2 transformation were used. A modified z-score transformation was applied to standardize the expression levels of surface markers across eight independent human colon cancer cell lines. As modified z-score varies between -3.5 and 3.5, this approach enabled the robust computation of each cell line’s capacity to polarize macrophages toward the M1 or M2 phenotype. Modified z-score is calculated using the formula z=0.6745×(x_n_ – xã)/MAD, where x_n_ is the raw fluorescence intensity of a surface marker in a certain cell line, xã is the median of surface marker fluorescence intensity across eight cell lines, MAD is the median absolute deviation. This method provides a robust standardization approach, minimizing the influence of outliers (25) Log2 transformation, calculated by applying the binary logarithm to a fold change in fluorescence intensity of a surface marker, was used to compare treatment conditions within a certain cell line.

### 2.7 Cytokine array for immune-related protein profiling

Cancer cell lysates were prepared by detaching cells with Trypsin/EDTA, suspending them in PBS, and then lysing them using T-PER Tissue Protein Extraction Reagent (Thermo Fisher Scientific, cat. no. 78510), supplemented with a protease/phosphatase inhibitor cocktail (Thermo Fisher Scientific, cat. no. EO0491). The lysates were centrifuged for 15 minutes at 10,000 x g at 4˚C, and the supernatants were collected while debris was removed. The protein concentration of each sample was measured using the Bradford assay. A C-1000 human cytokine immunoblot array (RayBio, cat. no. AAH-CYT-1000-8) was used to detect cytokines. First, 2 mL of blocking buffer was added to membranes, each placed in individual wells pre-coated with antibodies targeting the cytokines of interest. The membranes were incubated for 30 minutes at room temperature. After aspirating the blocking buffer, 1 mL of 5 mg/mL of each cell lysate was added per well and incubated overnight at 4°C. Following lysate incubation, the membranes were washed three times with two different washing buffers provided in the kit. Next, 1 mL of biotinylated antibody solution was added to each membrane and incubated for 2 hours at room temperature. The solution was aspirated, and the membranes were washed again with both washing buffers. Subsequently, 2 mL of HRP-Streptavidin solution was added and incubated for another 2 hours at room temperature, followed by a repeat washing process. The membranes were then transferred to a flat surface, and a detection buffer mixture was applied for 2 minutes. Immediately after incubation, the membranes were imaged using a C-DiGit chemiluminescence capture device.

The resulting data were analyzed using ImageJ software with the Protein Array Analyser plugin (26) to extract the integrated signal intensity values for each membrane spot. To visualize and compare semi-quantitative protein levels across eight independent human colon cancer cell lines, a modified z-score transformation was applied (as described in section 2.6), normalizing the raw signal intensity values by centering them around the median intensity and scaling by the median absolute deviation across all cell lines.

### 2.8 Cytotoxicity assay

To evaluate the cytotoxicity of stemness inhibitors *in vitro*, we used CCK-8 assay (Dojindo, cat. no. CK04-11). This assay measures cell viability by detecting the reduction of a tetrazolium salt by cellular dehydrogenases into a soluble orange formazan product, which is directly proportional to the number of metabolically active cells. CRC cells were seeded in 96-well plates at a density of 1×10^4 cells per 100 μL of RPMI medium. The plates were incubated for 24 hours at 37°C in a 5% CO_2_ atmosphere. Following incubation, 100 µL of varying concentrations of the tested inhibitors in RPMI medium were added to the wells: salinomycin (0.01, 0.1, 1, 10, 100 μM), SB-431542 (0.1, 1, 10, 100, 1000 μM), napabucasin (0.01, 0.1, 1, 10, 100 μM), and JIB-04 (0.01, 0.1, 1, 10, 100 μM). The cells were exposed to the drugs and cultured for 48 hours. After this, 200 µL of CCK-8 reagent mixed with RPMI medium was added to each well, followed by a 1-hour incubation. RPMI medium without cells was used as the control. CCK-8 absorbance was measured at 450 nm using a Biotek ELx800 microplate reader. The obtained data were analyzed using Gen5 software and Microsoft Excel. The inhibitory concentrations of the compounds that inhibited 50% of cell metabolic activity (IC50 values) were calculated from dose-response curves.

### 2.9 Effect of stemness inhibitors on CRC cell lines and macrophages *in vitro*

The impact of stemness inhibition on cell surface marker expression was evaluated *in vitro* using CRC cells, macrophages, and their co-cultures, prepared as described in sections 2.4 and 2.5. Cells were treated with the inhibitors as monotherapy or in combination, with concentrations as follows: 0.5 μM salinomycin, 10 μM SB-431542, 0.01 μM JIB-04, and 0.01 μM napabucasin. After 48 hours treatment, flow cytometry analysis was performed as described in section 2.6.

### 2.10 *In vivo* testing of stemness inhibitors in mouse breast cancer model

The syngeneic EO771 mouse breast cancer model, which expresses the stemness marker CD44 and generates a macrophage-rich immune tumor microenvironment (27, 28), was used to evaluate the *in vivo* effects of stemness inhibitors. For tumor implantation, female C57BL/6 mice (n=18, 8–12 weeks old) were anesthetized with 2% isoflurane (Vetpharma, cat. no. 90882) and injected subcutaneously in the right flank with 1 × 10^6 EO771 cells (>95% viability) suspended in 100 μL of PBS. After a 10-day period for tumor establishment, the mice received three doses of the specified drugs, either as monotherapy or in quadruple combination (n=3 per group), administered every three days. The following doses were used for the treatments: salinomycin (5 mg/kg), SB-431542 (10 mg/kg), JIB-04 (110 mg/kg), and napabucasin (20 mg/kg). All drugs were administered via intraperitoneal injection. Mouse were monitored for overall health and body weight. One day after the last dose, the mice were sacrificed in a CO_2_ chamber, and the tumors were collected for bulk RNA-seq analysis.

### 2.11 Transcriptomic analysis

Tumor samples were immediately processed upon collection. Up to 30 µg of tumor tissue was mechanically dissociated in TRIzol (Invitrogen, cat.no 15596026), and RNA was isolated using the RNeasy Mini Kit (Qiagen, cat. no. 74106) following the manufacturer’s instructions. RNA quantity and quality were assessed using a NanoDrop 2000 spectrophotometer.

Tumor RNA samples were processed using the Bulk RNA Barcoding and Sequencing (BRB-seq) platform with the Mercurius BRB-seq kit (Alithea Genomics, v.0.1.61, cat. no. 10813). Approximately 50 ng of total RNA was used per sample to generate BRB-seq libraries, following the manufacturer's protocol (29). Libraries were sequenced on an Illumina platform to a depth of approximately 8 million raw reads per sample. For the sequencing data analysis, raw reads were demultiplexed using STARsolo pipeline and quality-checked using FastQC (sequence quality, length, GC content). Barcodes and reads with a Phred quality score below 30 were trimmed using Cutadapt, and reads shorter than 20 nucleotides after trimming were discarded. High-quality reads were aligned to the *Mus musculus* genome (GRCm38) using STAR aligner with default parameters, resulting in 75.7% alignment rate and an average of 17041 genes detected per sample. Uniquely mapped reads were retained for downstream analysis. Transcript abundance was calculated using the transcripts per million (TPM) method with featureCounts v.2.0.3. Benchmarking included genome-wide Pearson correlation for quality control across samples.

To analyze the relative proportions of myeloid immune cell populations within the tumor microenvironment, we performed immune cell deconvolution using ImmuCellAI-mouse (30). This tool employs a gene signature-based hierarchical three-layers approach to estimate the infiltration levels of 36 immune cell subtypes, including various myeloid populations such as macrophages, dendritic cells (DCs), and neutrophils. For the deconvolution analysis, genes with low expression (TPM <1) were excluded to reduce noise. TPM values were uploaded to the online platform (31) and after processing, we obtained estimated infiltration scores for different immune cell types across all experimental groups. The results were analyzed to compare immune cell fractions between treatment groups and control, focusing on shifts in macrophage polarization and other myeloid populations.

### 2.12 TCGA analysis

The transcriptomic data of TCGA-COAD cohort (32) was retrieved using the cBioPortal web API on October 7, 2023. Upon retrieval, we curated the dataset to include only samples with full clinical annotations and RNA-Seq data. To quantify the proportions of immune cell subpopulations within the tumor microenvironment, we utilized the TIMER2.0 tool (33), which deconvolutes bulk RNA-Seq data to infer the abundance of various immune cell types. For the classification of the colorectal cancer samples into the Consensus Molecular Subtypes, we downloaded the CMS annotations from SYNAPSE Colorectal Cancer Subtyping Consortium (34). Additionally, for survival prediction analysis based on cytokine expression profile, SurvExpress was employed (35).

### 2.13 Statistical analysis

Data were analyzed using GraphPad Prism 9 (GraphPad Software, USA). For each experiment, the averages of four independent replicates were evaluated. Heatmaps displaying the protein expression profiles were generated using Morpheus software (Broad Institute) (36), where z-transformed or log2-transformed expression values were used for relative protein levels in lysates or median fluorescence intensity-derived expression levels of surface markers.

Data in bar graphs are presented as medians with interquartile range. Statistical significance for multiple group comparisons was determined using a one-way ANOVA followed by *post hoc* testing, while the Mann-Whitney U test was applied for comparisons between two groups. Correlations between variables were assessed using Pearson correlation, with a 95% confidence interval. Chi square test was used to assess the association between categorical variables. A significance threshold was set at *p* < 0.05, and was tested for false discovery rate (FDR) correction with an alpha of 10%.

## 3 Results

### 3.1 Consensus molecular subtype-associated macrophage polarization patterns *in vitro*

The tumor microenvironment plays a crucial role in shaping immune responses, particularly through interactions with macrophages. In this study, we used eight molecularly diverse colorectal cancer cell lines (COLO320, DLD1, LoVo, LS1034, NCI-H508, HT29, HCT116, SW620) representing different CMS (9, 37) to modulate macrophage polarization, aiming to identify subtype-specific immune-modulatory patterns. Macrophages derived and differentiated from human PBMC monocyte fraction were co-cultured with colorectal cell lines for 48 hours in an indirect transwell system and then analyzed by flow cytometry for surface marker expression.

**Figure 1:**
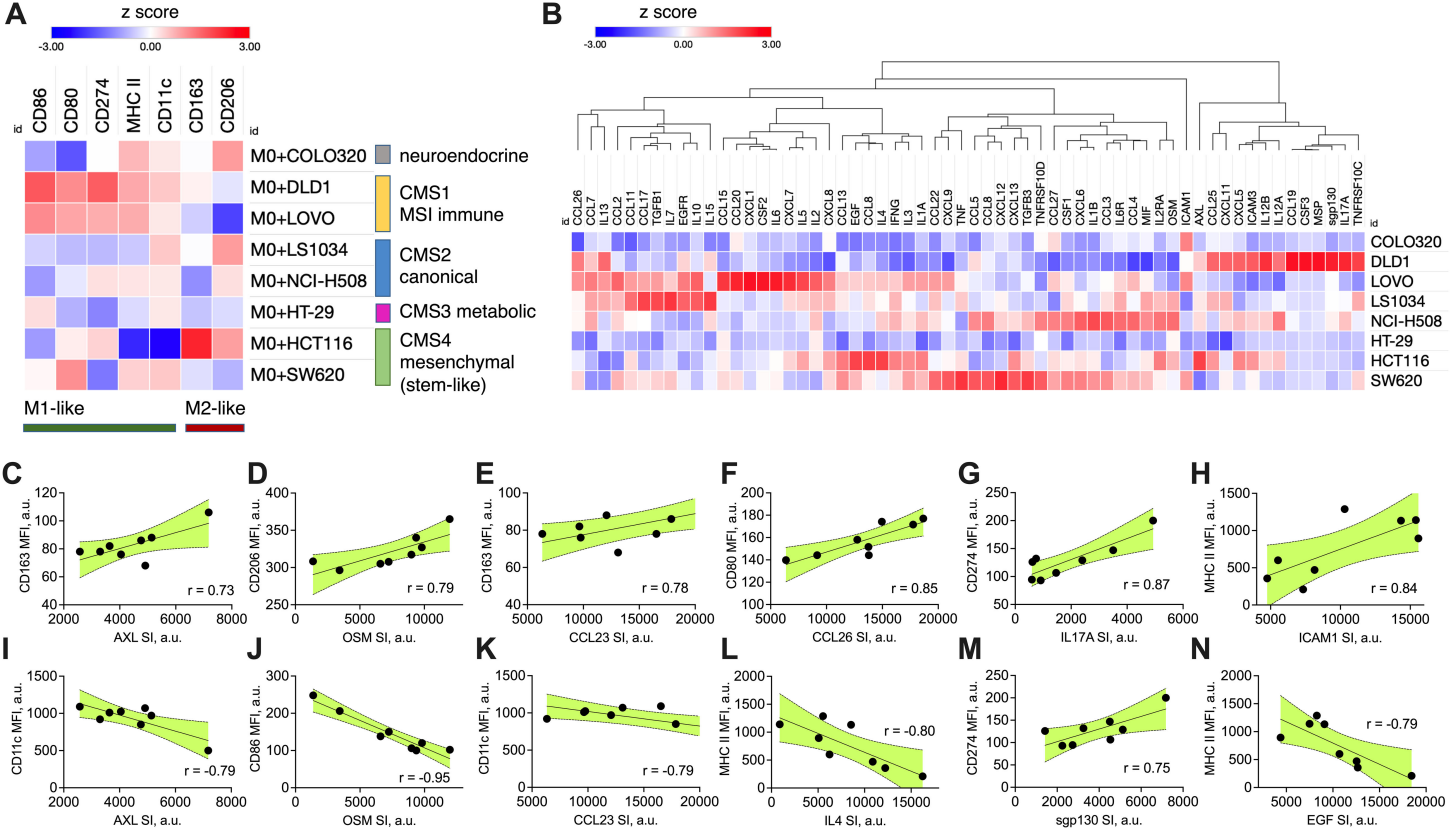
CRC-induced macrophage polarization and its association with specific cytokine production signatures. **(A)** Heatmap showing the marker expression on M0 macrophages after 48-hour transwell co-culture with CRC cell lines of different molecular subtype (CMS 1-4). Macrophage polarization was assessed by flow cytometry using specific M1 (CD86, CD80, CD274, MHC II, CD11c) and M2 (CD163, CD206) markers. Data are presented as modified z-scores of MFI, with red indicating high expression and blue indicating low expression. **(B)** Hierarchical clustering heatmap of cytokine and chemokine production profiles from CRC cell lines, quantified using semi-quantitative membrane-based cytokine array. The relative protein expression in cell lysates is shown as modified z-scores, with red indicating high expression and blue indicating low expression. **(C–N)** Correlation plots showing cancer cell cytokine production (X axis, SI values) against macrophage marker expression (Y axis, MFI values). Pearson correlation coefficients (r) are displayed in each plot, with shaded areas representing the 95% confidence interval. All shown correlations were statistically significant (two-sided p < 0.05, Pearson correlation test), indicating strong positive or negative associations between specific cytokines and macrophage polarization markers. MFI – median fluorescence intensity, CMS – consensus molecular subtype, SI – signal intensity, a.u. – arbitraty units.

We quantified macrophage polarization strength based on the individual expression of key M1 (CD86, CD80, MHC II, CD274, CD11c) and M2 (CD163, CD206) markers. The co-culture results demonstrated that the cancer cell lines induced distinct macrophage phenotypes (**Figure 1A**). To robustly evaluate the macrophage polarization potential across CRC cell lines, we first calculated the modified z-scores for each marker, showing how far the marker fluorescence intensity value of each cell line deviates from the median of all cell lines, expressed in terms of median absolute deviations. Then, for each colorectal cancer cell line, an average of marker z-scores was calculated for both M1 and M2 markers. These scores were used to classify the macrophage polarization type as either M1, M2, mixed, or unpolarized, depending on the relative dominance of M1 and M2 scores. The polarization strength was categorized as none (z-scores between -0.5 and 0.5), moderate (0.5 to 1), or strong (z-scores > 1). The macrophage polarization potential for each CRC cell line is summarized in **Table 2**.

**Table 2:** Polarization scores and protein expression profiles of CRC cell lines based on M1 and M2 macrophage markers.

Cell line	CMS subtype (37)	M1 score	M2 score	Polarization type	Polarization strength	Top expressed proteins
COLO320	NE	-0.46	0.48	Mixed	Moderate	ICAM1
DLD1	CMS1	1.31	-0.06	M1	Strong	CSF3, MSP, CCL19, sgp130, IL17A, IL12B, TNFRSF10C, ICAM3, CXCL5, CXCL11
LoVo	CMS1	0.89	-1.16	M1	Moderate	CXCL1, CSF2, IL6, CCL20, CXCL7, CCL15, IL5, IL2, IL10, CCL2
LS1034	CMS2	-0.23	0.47	Mixed	Moderate	IL7, IL15, CCL17, TGFB1, IL10, EGFR, CCL11
NCI-H508	CMS2	-0.17	-0.39	Unpolarized	None	IL1B, CCL3, CXCL6, CCL4, IL6R, OSM
HT-29	CMS3	-0.34	-0.45	Unpolarized	None	IL2
HCT116	CMS4	-0.63	1.61	M2	Strong	CCL8, AXL, IL4, EGF, CCL13
SW620	CMS4	0.58	-0.51	M1	Moderate	CXCL12, TNF, TGFB3, CCL8, CCL5, CXCL9, CXCL13, TNFRSF10D, CCL22, CSF1

M1 and M2 polarization scores were calculated by averaging z-scores for respective M1 (CD86, CD11c, CD80, MHC II, CD274) and M2 (CD163, CD206) markers. Based on these scores, the polarization type was determined: M1 (if M1 score > 0.5 and M2 < 0), M2 (if M2 score > 0.5 and M1 < 0), Mixed (if neither score exceeded ±0.5), or Unpolarized (if both scores were < 0). Polarization strength was classified as none (if the score is less than 0.5), moderate (0.5 to 1), or strong (above 1).

CMS, Consensus Molecular Subtype; NE, neuroendocrine.

DLD1 and LoVo, classified as CMS1, induced M1-like polarization, marked by elevated surface expression of M1-like markers (MHC II, CD86) and reduced M2-like markers (CD206). This supports the immune-active profile of CMS1 tumors, known for higher immune infiltration. CMS2 (LS1034, NCI-H508) and CMS3 (HT29) lines induced more balanced macrophage polarization, with weak M1 and M2 marker expression, while COLO320, lacking a clear CMS classification, showed minimal polarization, altogether indicating weak immune modulation across these subtypes. HCT116 and SW620, both classified as CMS4, displayed divergent effects on macrophage polarization. HCT116 induced strong M2-like macrophage polarization (increase in M2-associated surface markers such as CD163 and CD206, low M1-like markers). SW620, however, induced a moderate M1-like polarization with an increase in M1 markers (CD80, MHC II and CD11c). This mixed polarization potential reflects the complex immune microenvironment often observed in mesenchymal tumors.

The macrophage polarization profiles were further validated by comparing the relative proportion of macrophages in the CMS subtypes of TCGA CRC patient cohort (TCGA-COAD), as deconvoluted using TIMER2.0. The overall proportion of macrophages was highest in CMS4 tumors compared to other subtypes (p<0.05) (**Supplementary Figure 1A**). The M1/M2 ratio was highest in CMS1 tumors, with CMS4 tumors significantly lower than CMS1 (p<0.05) (**Supplementary Figure 1B**). CMS1 tumors had the highest relative proportion of M1 macrophages, followed by CMS4, while CMS3 had the lowest (**Supplementary Figure 1C**). Finally, CMS4 tumors exhibited the highest proportion of M2 macrophages (p<0.05) (**Supplementary Figure 1D**). Although CMS did not seem to affect patient outcomes in TCGA cohort (**Supplementary Figure 1E**), the above observations confirm the immune-suppressive nature of mesenchymal-like CMS4 tumors and highlight the need for further investigations into their macrophage-modulating mechanisms and myeloid-based targeting strategies.

To further investigate the association between cancer cell-induced macrophage polarization and cytokine expression, we quantified the immune-related protein profiles in each cell line using a semi-quantitative cytokine proteome profiler (**Figure 1B**). For comparison, relative protein expression levels extracted from immunoblot were standardized across eight independent human colon cancer cell lines using modified z-score transformation, reflecting how far the relative protein signal intensity in each cell line deviates from the median of all cell lines, expressed in terms of median absolute deviations. The top expressed proteins for each cell line are summarized in **Table 2** and include cytokines, chemokines, growth factors, adhesion molecules, and receptors. Most cell lines expressed a mix of both pro-inflammatory and anti-inflammatory factors. The expression signature of HCT116 cells predominantly indicated an anti-inflammatory origin (e.g., CCL8, AXL, IL4, EGF, CCL13), aligning with their capacity to induce M2-like macrophage polarization. In contrast, the SW620 cell line exhibited a mixed phenotype, with pro-inflammatory (potential M1-like modulators: TNF, CXCL9, CXCL13) and anti-inflammatory factors (potential M2-like modulators: TGFB3, CCL8, CCL22, CSF1), reflecting its capacity to induce mixed macrophage polarization profiles.

Statistical analysis across all cell lines revealed several significant correlations between cytokine expression and macrophage polarization (**Figures 1C–N**). CCL26 (**Figure 1F**), IL17A (**Figure 1G**), sgp130 (IL6ST) (**Figure 1M**), and ICAM (**Figure 1H**) positively correlated with M1-like macrophage surface marker induction, suggesting their potential role in promoting anti-tumoral immune responses. AXL (**Figures 1C, I**), OSM (**Figures 1D, J**), CCL23 (**Figures 1E, K**), IL4 (**Figure 1L**), and EGF (**Figure 1N**) positively correlated with M2-like macrophage surface marker induction or negatively correlated with M1-like macrophage surface marker induction, highlighting their potential role in supporting pro-tumoral immune responses. Combining these nine proteins signatures into a prognostic marker set using SurvExpress stratified TCGA-COAD cohort into high risk and low risk groups with distinct survival outcomes (p=0.003, **Supplementary Figure 2**). This suggests a potential of a composite biomarker signature to capture the moderate association (concordance index 61.86) between cytokine expression and patient prognosis.

Additionally, given the role of stemness in tumor aggressiveness, we explored whether the stemness phenotype of the CRC cells, defined by CD44 and CD133 surface markers expression, influenced macrophage polarization. Using flow cytometry, we examined the proportions of CD44+ and CD133+ in cancer cells (**Supplementary Figures 3A-H**, quadrant gating on live cells, gate thresholds set accounting for single stainings). COLO320 (**Supplementary Figure 3A**), NCI-H508 (**Supplementary Figure 3G**), and SW620 (**Supplementary Figure 3H**) were characterized with low (<10% of marker-positive cells) expression of both CD44 and CD133. DLD1 (**Supplementary Figure 3B**) and LS1034 (**Supplementary Figure 3F**) expressed CD44 (42% and 12% of marker-positive cells, respectively), but not CD133. HCT116 (**Supplementary Figure 3C**), HT29 (**Supplementary Figure 3D**), and LoVo (**Supplementary Figure 3E**) strongly (>40% of marker-positive cells) expressed both CD44 and CD133. Although there was variation between CRC cell lines of the same subtype, the association between CMS subtype and stemness marker expression (p=0.020, chi-square test) revealed that CMS1 and CMS4 subtypes are characterized with high (>40%) expression of CD44 and moderate-to-low (<25%) expression of CD133. Moreover, when stemness marker CD44 and CD133 expression compared with the capacity to induce macrophage phenotype in transwell co-culture (**Figure 1A**), we observed a strong correlation between the size of the CD44+/CD133- subpopulation in CRC cell lines and the induction of M2-like macrophage marker CD163 (**Supplementary Figure 3I**) together with downregulation of M1-like marker CD11c (**Supplementary Figure 3J**). The proportion of CD44+/CD133- cell subset in CRC cells positively correlated with the expression of CD274 (PD-L1) on macrophages (**Supplementary Figure 3K**). This supports our hypothesis that pronounced stemness properties of cancer cells are associated with their ability to promote the M2-like polarization in macrophages.

Our findings demonstrate distinct macrophage polarization patterns across colorectal cancer cell lines, with CMS1 favoring M1-like polarization, CMS2 and CMS3 showing balanced profiles, and CMS4 lines (HCT116 and SW620) exhibiting divergent polarization. HCT116 promoted strong M2-like polarization, supported by an anti-inflammatory cytokine profile. In contrast, SW620 showed a mixed M1/M2 phenotype, with cytokine expression reflecting both pro- and anti-inflammatory factors. TCGA data further supported the high macrophage infiltration, predominantly M2-like, in CMS4 tumors. Based on these findings, CMS4 cell lines (HCT116 and SW620) emerged as key candidates for further investigation, particularly in targeting their stem-like properties.

### 3.2 Cytotoxicity of stemness inhibitors in colorectal cancer cell lines

Next, we aimed to investigate how inhibiting stemness-related pathways could influence both the stemness-associated phenotype of CMS4 CRC lines (HCT116 and SW620) and their capacity to modulate the tumor microenvironment, particularly macrophage polarization. Given the mesenchymal and stem-like characteristics of CMS4 tumors, we hypothesized that targeting stemness would not only directly affect cancer cells but also modulate key components of the tumor microenvironment, such as macrophages, potentially leading to broader impacts on immune modulation and tumor progression.

We used four inhibitors targeting different pathways involved in cancer stemness. Salinomycin is known to disrupt Wnt/β-catenin signaling, effectively reducing cancer stem cell populations (38, 39). SB-431542 inhibits the TGF-β receptor, which is critical for maintaining stemness and mesenchymal traits in tumor cells (40). JIB-04, a histone demethylase inhibitor, targets epigenetic regulators involved in stemness pathways (41), while napabucasin, a STAT3 inhibitor, blocks key transcriptional pathways that promote cancer stem cell survival (42, 43). These inhibitors were selected for their ability to target distinct but complementary pathways implicated in cancer stemness and tumor progression.

**Figure 2:**
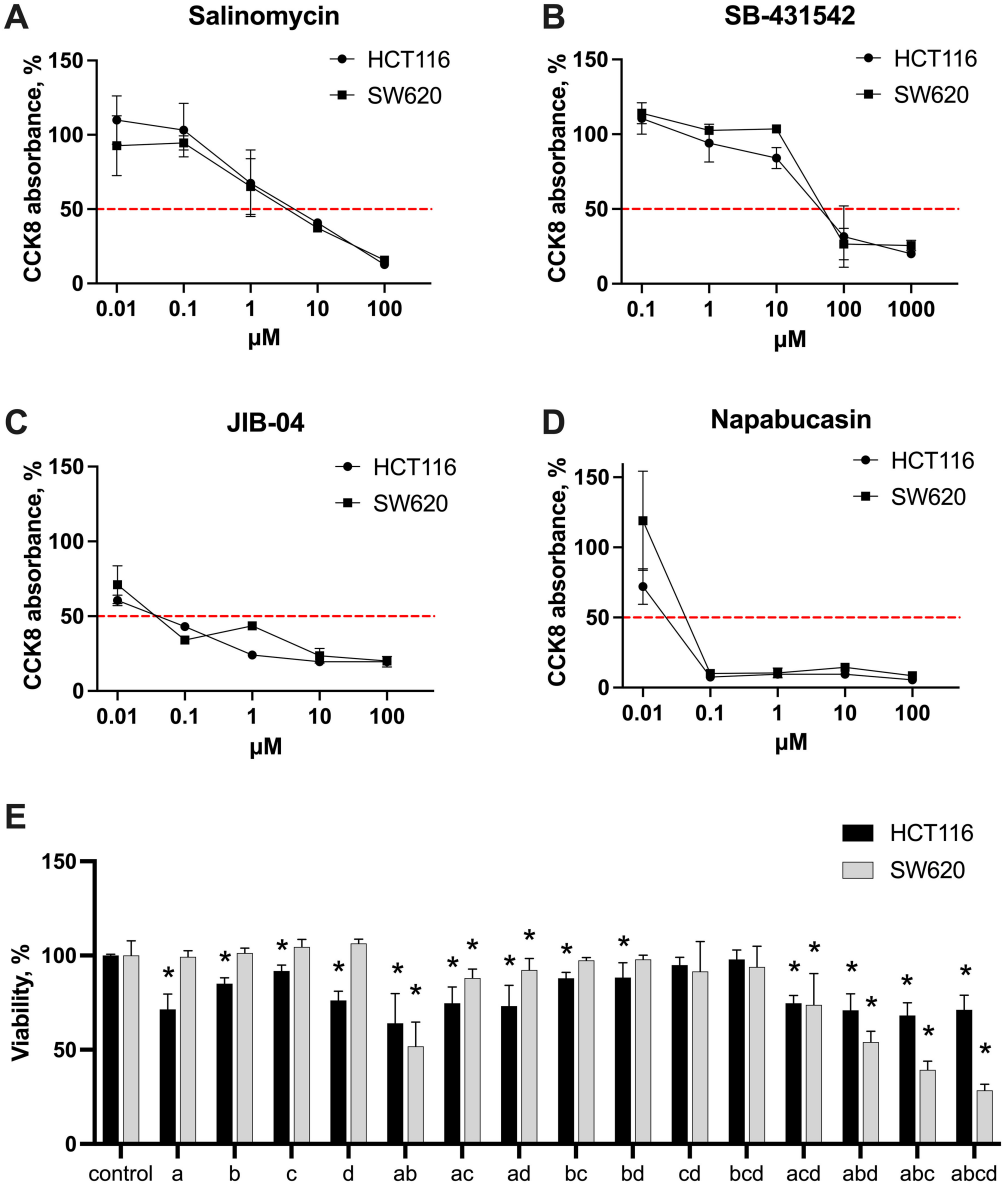
Cytotoxic effects of stemness inhibitors. Dose-response curves of monotherapy salinomycin **(A)**, SB-431542 **(B)**, JIB-04 **(C)**, napabucasin **(D)** across the range of 0.01-1000 μM in HCT116 and SW620 cells, measured with CCK-8 assay. The red dashed line indicates 50% of CCK-8 absorbance in untreated samples, used to extrapolate IC50 values. **(E)** The cytotoxic effects of the inhibitors—salinomycin **(A)**, SB-431542 **(B)**, JIB-04 **(C)**, napabucasin **(D)**—and their combinations in HCT116 and SW620 cells were evaluated using flow cytometry scatter analysis. Results are shown as median ± interquartile range from three independent experiments. Statistically significant differences compared to the untreated control are indicated by asterisks (*), where p < 0.05.

First, the dose-response experiments were conducted to estimate the IC50 values of each inhibitor in both CMS4 CRC cell lines HCT116 and SW620. Cells in the logarithmic growth phase were incubated under standard conditions for 48 hours with 0.01–100 μM for salinomycin, napabucasin, and JIB-04, and 0.1–1000 μM for SB-431542. Cytotoxicity was assessed using the CCK-8 assay, which measures metabolic activity and provides an indirect assessment of cell viability. IC50 values, were derived from dose-response curves at 50% of CCK-8 absorbance. The results revealed that both HCT116 and SW620 showed similar sensitivity to the tested inhibitors within the concentration range evaluated. The IC50 values were comparable for both cell lines: salinomycin – 3 µM for both (**Figure 2A**), SB-431542 – 60 µM for both (**Figure 2B**), JIB-04 – 0.04 µM for both (**Figure 2C**), and napabucasin – 0.02 µM in SW620 and 0.03 µM in HCT116 (**Figure 2D**). Among all the compounds, napabucasin demonstrated the most potent inhibition of metabolic activity (IC50 = 0.02–0.03 µM), while SB-431542 exhibited the weakest effect, with an IC50 value of 60 µM, approximately 2400 times higher than napabucasin. These findings highlighted the differing efficacy of stemness inhibitors in inducing cytotoxicity.

Based on the results of the monotherapy experiments and literature data, concentrations were selected for further combination therapy experiments: salinomycin (0.5 µM), SB-431542 (10 µM), JIB-04 (0.01 µM), and napabucasin (0.01 µM). To explore the effects of inhibitors on cell viability, both cell lines were treated with selected concentrations, alone and in combinations, and their viability was approximated via flow cytometry using scatter analysis (**Figure 2E**). Monotherapy results revealed no significant differences in sensitivity between HCT116 and SW620. However, in combination therapy, SW620 cells were more sensitive to triple and quadruple drug combinations compared to HCT116 cells. In contrast, HCT116 cells showed a more modest response to combination therapies. Based on these findings, we next assessed the phenotypic changes in both cancer cells and macrophages following treatment with stemness inhibitors alone or in combinations, aiming to better understand the broader impact of these therapies on the tumor microenvironment.

### 3.3 Effect of stemness inhibitors on surface phenotype of colorectal cancer cell lines

We further aimed to explore the effects of stemness inhibitors on the surface phenotype of CMS4 colorectal cancer cells, focusing on HCT116 and SW620 cell lines. For this, the growth medium was supplemented with salinomycin (0.5 μM), SB-431542 (10 μM), napabucasin (0.01 μM), JIB-04 (0.01 μM), and their quadruple combination for 48 hours. Afterwards, CRC cells were analyzed for the fold change in surface marker expression (CD44, CD133, ESA for stemness; MHC I, CD274 for immunomodulation) upon treatment, with each marker’s median fluorescence intensity measured individually by flow cytometry. Additionally, we used an indirect co-culture system to investigate the combined effects of these inhibitors on cancer cells in the presence of PBMC-derived macrophages.

**Figure 3:**
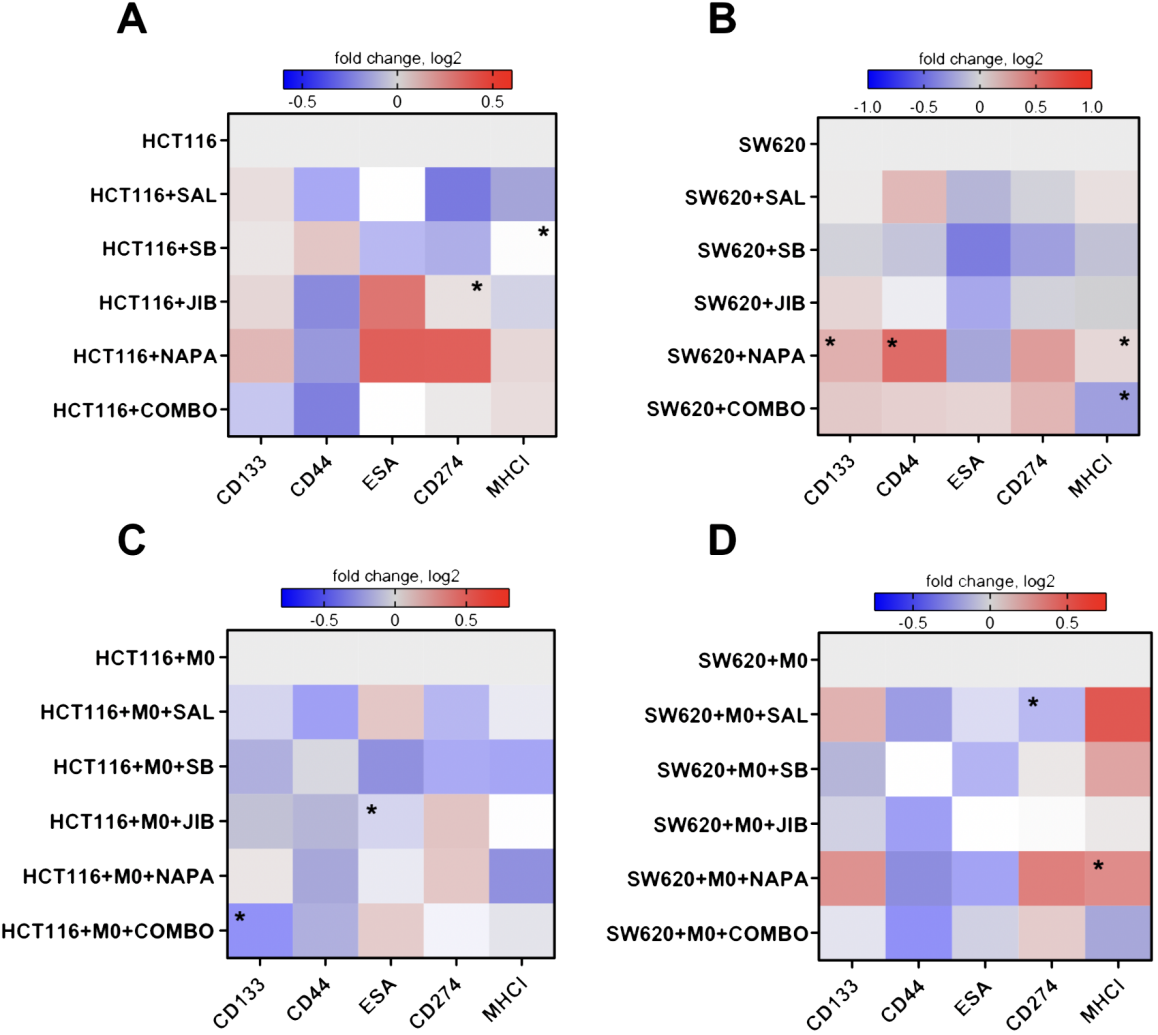
Effects of stemness inhibitors on stemness and immunomodulatory surface marker expression in colon cancer cells. Stemness inhibitors affect the phenotype of cancer cells. **(A, B)** Heatmap illustrating the changes in surface marker expression following monotherapy and combination therapy with stemness inhibitors (salinomycin, SB-431542, JIB-04, and napabucasin) in HCT116 and SW620 cells. **(C, D)** The effects of co-culture with M0-like macrophages on the expression of surface markers in HCT116 and SW620 cell lines treated with the same inhibitors. Transwell co-cultures were conducted for 48 hours, with medium supplemented with 0.5 µM salinomycin, 10 µM SB-431542, 0.01 µM JIB-04, 0.01 µM napabucasin, and a combination of these compounds. The expression of stemness (CD44, CD133, ESA) and immunomodulation (MHC I, CD274) markers was measured with flow cytometry. Log2 transformation was applied to the data, and color intensity represents changes in surface marker median fluorescence intensity, with red indicating an increase (higher log2 value) and blue indicating a decrease (lower log2 value) relative to control. Data are presented as medians from four independent experiments. Statistical significance compared to control samples is denoted by an asterisk (*) where p < 0.05.

The data suggest that the two cell lines, HCT116 and SW620, responded differently to the treatment regimens (**Figures 3A, B**). In HCT116 cells, exposure to single inhibitors—salinomycin and SB-431542—appeared to reduce the immune checkpoint marker CD274 (PD-L1), whereas napabucasin increased its expression. In terms of stemness markers, single inhibitors appeared to increase CD133 expression in HCT116 cells, while CD44 expression was reduced by salinomycin, JIB-04, and napabucasin. The combination of inhibitors downregulated both CD133 and CD44 in HCT116 cells. ESA expression, however, only showed a reduction in HCT116 cells treated with SB-431542.

For SW620 cells, the results differed (**Figure 3B**). Single inhibitors such as salinomycin, SB-431542, and JIB-04 led to a decrease in CD274 expression, while napabucasin and quadruple combination caused an increase. MHC I expression was reduced under combination treatment in SW620 cells. ESA expression was generally reduced across all single inhibitors. The treatment with napabucasin significantly increased the CD133, CD44, and MHC I surface expression in SW620 cells (p<0.05). Quadruple combination therapy significantly downregulated MHC I on SW620 cells (p<0.05).

When co-cultured with macrophages, HCT116 cells appeared to show a stronger reduction in stemness markers (**Figure 3C**) upon targeting. Both single inhibitors and combination therapy resulted in decreased CD133 and CD44 expression. Similarly, in SW620 cells, CD44 expression was reduced across treatments, and SB-431542, JIB-04, and combination therapy downregulated CD133 (**Figure 3D**). ESA marker expression was consistently reduced by SB-431542, JIB-04, and napabucasin in both cell lines. Regarding immunomodulatory effects, in HCT116 cells, salinomycin, SB-431542, and combination therapy reduced CD274 expression. However, all inhibitors also reduced MHC I expression to some extent. Conversely, in SW620 cells, salinomycin, SB-431542, and napabucasin (p<0.05) appeared to promote MHC I expression. CD274 expression was significantly downregulated upon treatment with salinomycin (p<0.05).

Our findings demonstrate that stemness inhibitors impact both stemness and immune markers in CMS4 colorectal cancer cell lines, with HCT116 and SW620 showing distinct responses. In HCT116, combination therapies, especially salinomycin and SB-431542, strongly downregulated CD44 in both monoculture and co-culture with macrophages. CD133 expression was also reduced, but only in co-culture conditions with macrophages. In SW620, the decrease in CD44 was observed only in co-culture with macrophages, but not in monoculture, indicating that the presence of macrophages may enhance the sensitivity of SW620 cells to stemness inhibition. This suggests that the tumor microenvironment, and specifically interactions with macrophages, plays a crucial role in modulating the response of cancer stem-like properties to treatment.

Immune-related markers also showed divergent trends upon CRC co-culture with macrophages: MHC I was reduced in HCT116 following treatment, potentially lowering immune recognition, while in SW620, MHC I increased, suggesting enhanced immune surveillance. Furthermore, CD274 was reduced by salinomycin in both cell lines, indicating a possible immune-stimulatory effect. Having observed distinct responses in CMS4 CRC cell lines to stemness inhibitors, we next aimed to explore the effects of these treatments on macrophages, the most abundant immune cells within the tumor microenvironment, to determine their broader impact on TME-associated immune modulation.

### 3.4 Effect of stemness inhibitors on macrophage surface phenotype

Given the central role of macrophages in shaping the tumor microenvironment (TME) and their plasticity across a spectrum of activation states—from pro-inflammatory (M1-like) to anti-inflammatory (M2-like)—we explored whether stemness inhibitors, originally designed to target cancer cells, could also modulate macrophages as an additional therapeutic strategy. We examined the response of PBMC-derived macrophages to stemness inhibitors under two conditions: direct treatment or in co-culture with CMS4 CRC cell lines HCT116 and SW620. To assess macrophage polarization, the growth medium was supplemented with 0.5 μM salinomycin, 10 μM SB-431542, 0.1 μM JIB-04, 0.1 μM napabucasin, or a quadruple combination of these inhibitors. Flow cytometry was used to analyze fold changes in median fluorescence intensity of individual surface markers associated with M1-like (CD86, CD80, CD11c, MHC II, CD274) and M2-like (CD163, CD206) macrophage phenotypes.

**Figure 4:**
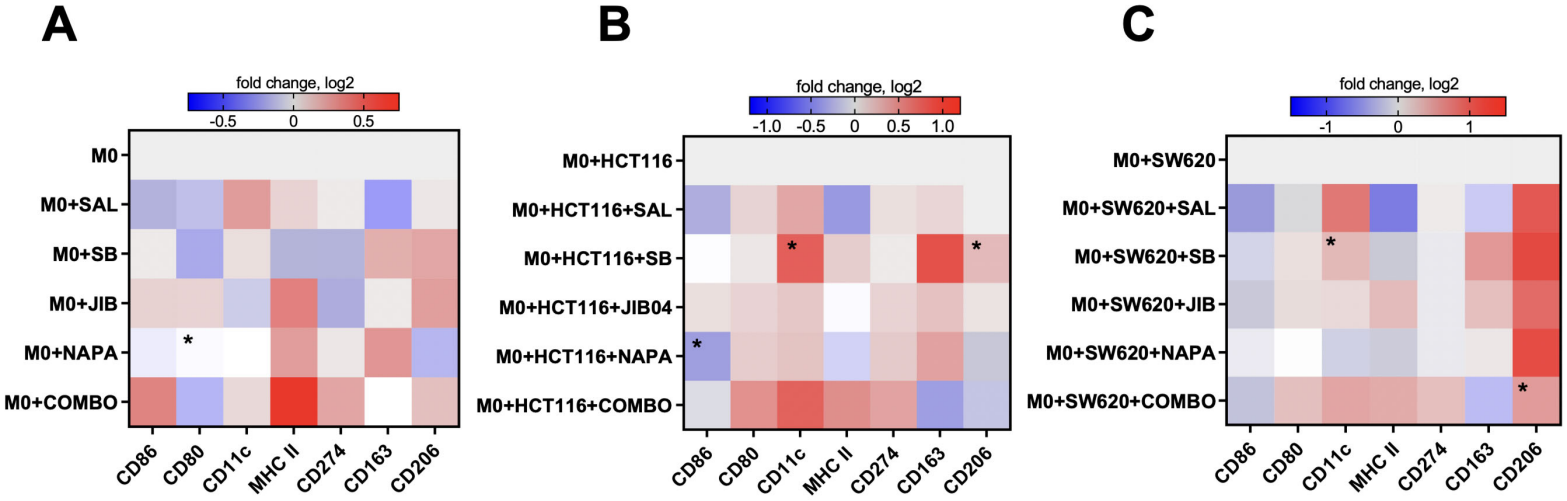
M1-like and M2-like surface marker expression profiles in macrophages upon treatment with stemness inhibitors. Stemness inhibitors induce changes in M1- and M2-related phenotype in M0-like macrophages **(A)**. Co-culture with different tumor cell lines (HCT116 and SW620) in combination with stemness inhibitors treatment leads to further alterations in M0-like macrophage polarization **(B, C)**. PBMCs were differentiated into M0 macrophages using 20 ng/ml M-CSF over six days. The expression of M1 (CD86, MHC II, CD80, CD274, CD11c) and M2 (CD163, CD206) surface markers was analyzed by flow cytometry after 48 hours of co-culture with HCT116 and SW620 cells in medium supplemented with 0.5 µM salinomycin (SAL), 10 µM SB-431542 (SB), 0.01 µM JIB-04 (JIB), 0.01 µM napabucasin (NAPA), or a combination of these compounds. Heatmaps represent the log2-transformed median fluorescence intensity of surface marker expression in M0-like macrophages following drug exposure **(A)** and after co-culture with tumor cells and drug treatments **(B, C)**. Color bars indicate the magnitude of expression, with red denoting higher expression and blue indicating lower expression. Data are representative of four independent experiments and are displayed as median. Statistical significance compared to control samples is denoted by an asterisk (*) where p < 0.05.

The results showed variable effects of individual inhibitors on M0 macrophages, with no consistent trend toward M1 polarization (**Figure 4A**). Salinomycin, JIB-04, and napabucasin increased MHC II expression, while CD80 and CD274 expression decreased across all single-inhibitor treatments, suggesting a reduction in immunosuppressive activity. For M2 markers, salinomycin reduced CD163 expression, and napabucasin downregulated CD206, indicating potential shifts away from M2-like polarization. The quadruple drug combination had a more pronounced effect, increasing the expression of CD86, MHC II, and CD11c, while downregulating CD163, signaling a shift toward an M1-like phenotype.

In the HCT116 co-culture, single inhibitors selectively modulated M2-associated markers without consistent changes in M1 markers (**Figure 4B**). Salinomycin and napabucasin downregulated CD86 (p<0.05 for napabucasin) and MHC II, while SB-431542 significantly increased CD11c and CD206 expression (both p<0.05). The quadruple combination had a more comprehensive effect, reducing CD163 and CD206 expression while increasing CD86, CD80, MHC II, and CD11c, suggesting a shift toward an M1-like phenotype that counteracted the M2-like state induced by HCT116 cells.

In the SW620 co-culture, most inhibitors as monotherapies decreased CD86 and MHC II expression while increasing CD206 (**Figure 4C**). SB-431542 notably increased CD11c expression. The quadruple combination had a more pronounced impact, reducing CD163 and increasing CD80, CD11c, and MHC II, indicating a shift toward an M1-like state. However, CD206 remained upregulated, indicating a mixed macrophage phenotype with some persistent M2-like features.

Overall, these findings suggest that stemness inhibitors, particularly in combination, can modulate macrophage polarization toward an M1-like phenotype, especially in the HCT116 co-culture. Single inhibitors produced more variable and context-dependent effects, while the quadruple combination consistently promoted an M1-like state by upregulating pro-inflammatory markers (CD86, HLA-DR, CD11c) and downregulating M2 markers (CD163, CD206). These effects were more prominent in co-culture, indicating that the presence of CRC cells modulates macrophage responses to stemness inhibitors. This highlights the potential of using stemness-targeting therapies not only to target cancer cells but also to reprogram the TME by shifting macrophage polarization.

### 3.5 Treatment with stemness inhibitors modulates the myeloid immune tumor microenvironment in an *in vivo* tumor model

While our *in vitro* studies centered around colorectal cancer, specifically the CMS4 subtype, we extended our research to an *in vivo* model to investigate how stemness inhibitors might perturb the immune landscape of the tumor microenvironment. Given that key stemness-related pathways—such as Wnt/β-catenin, TGF-β, and STAT3—are conserved across various tumor types, including colorectal and breast cancers (44–46), we conducted an *in vivo* study of stemness inhibitors using the EO771 triple-negative breast cancer mouse model. Due to mesenchymal traits characteristic to TNBC tumors (22), self-renewal potential of the EO771 cell line (47) the abundance of macrophages in the microevironment of syngeneic EO771 tumors in immunocompetent mice (27, 28) this model emerged as relevant system for investigating the interplay between stemness inhibition and immune modulation within a mesenchymal tumor microenvironment.

To confirm the stemness and immunomodulation markers expression on mouse breast cancer cell lines, we analyzed the expression of CD44 and CD274 (PD-L1) with flow cytometry in two cell lines, EO771 and 4T1 (**Supplementary Figure 4**). EO771 cell line was characterized with high expression of CD44 (96% of marker-positive cells, p=0.056 compared to 89% in 4T1) and CD274 (68% of marker-positive cells, p<0.05 compared to 39% in 4T1), reflecting its stemness and immunosuppressive potential.

Ten days after subcutaneous injection of 1 million of EO771 cells into 8-12 week C57BL/6 female mice right flank, tumors reached around 100-200 mm³. Each treatment group (n=3 per group) received three doses of stemness inhibitors (every 3 days) in monotherapy or combination therapy. Tumors were harvested one day after the last treatment. To assess the immune modulation induced by stemness inhibitors, we used a deconvolution approach on RNA-seq data to analyze the relative proportions of myeloid immune cells within the tumor. Treated tumors were compared against the untreated control group.

**Figure 5:**
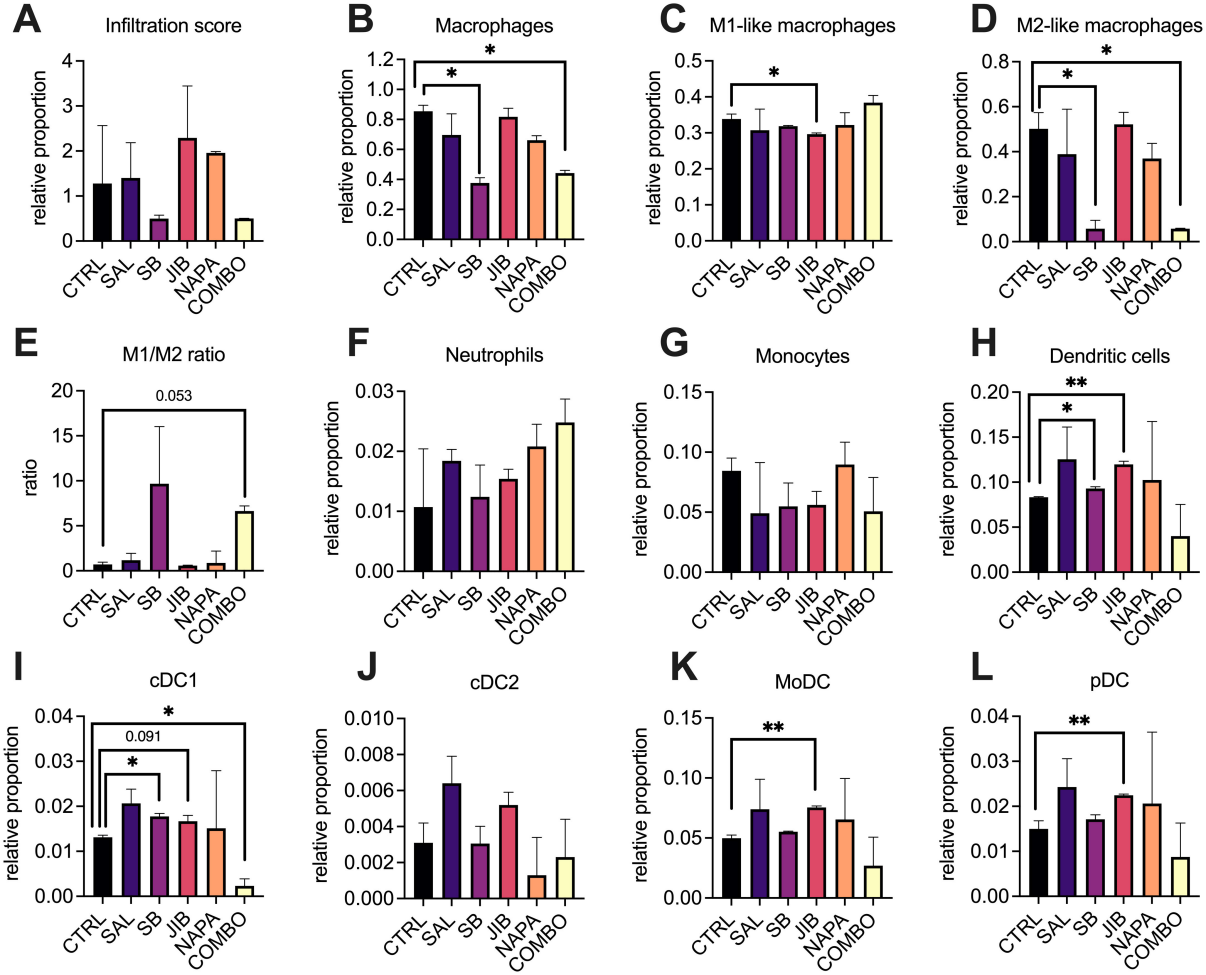
Deconvolution of myeloid cells profiles in the tumor microenvironment after stemness inhibition *in vivo*. EO771 tumors were collected after monotherapy or combination treatment (n=3 per group) with stemness inhibitors (salinomycin 5 mg/kg, SB-431542 10 mg/kg, JIB-04 110 mg/kg, napabucasin 20 mg/kg) and subjected to RNA-sequencing using the BRB-seq platform. The immune composition of the tumor microenvironment was then deconvoluted using ImmuCellAI-mouse, revealing the relative proportions of immune cell populations. Each bar represents the relative infiltration score of different immune cell types in treated and control tumors. Immune infiltration score **(A)** as well as relative proportion of distinct immune cell subtypes such macrophages **(B)**, M1 **(C)** and M2 **(D)** macrophages, their ratio **(E)**, neutrophils **(F)**, monocytes **(G)**, DCs **(H)**, including conventional DCs type 1 **(I)**, conventional DCs type2 **(J)**, monocytic DC **(K)** and plasmacytoid DC **(L)** are shown. Comparisons between treatment groups and control highlight shifts in immune cell proportions following treatment with stemness inhibitors. All data are shown as median ± interquartile range. Statistical significance compared to control samples is denoted by an asterisk (*) where p < 0.05, (**) where p < 0.01. M – macrophage, DC – dendritic cell, CTRL – untreated control, SAL – salinomycin monotherapy, SB - SB-431542 monotherapy, JIB - JIB-04 monotherapy, NAPA – napabucasin monotherapy, COMBO – quadruple combination therapy group.

The results showed no significant changes in overall immune infiltration when comparing the treated groups with controls, indicating that stemness inhibitors did not drastically alter the total immune cell presence in the tumor (**Figure 5A**). However, interesting shifts in the relative proportion of macrophages (**Figures 5B–E**) and dendritic cells (**Figures 5H–L**) were observed. The relative proportion of macrophages decreased at least two-fold in tumors of mice treated with SB-431542 and quadruple combination in comparison with untreated tumors (p<0.05 for both) (**Figure 5B**). Although the M1 macrophage proportion remained mostly stable, except for minor yet statistically significant (p<0.05) decrease in tumors treated with JIB-04 (**Figure 5C**), the more dramatic reduction was observed in relative proportion of intratumoral M2 macrophages in mice treated with SB-431542 and quadruple combination, compared with untreated tumors (p<0.05 for both) (**Figure 5D**). Altogether, these perturbations resulted in positive shift of M1/M2 ratio seen in both the SB-431542 and quadruple combination treatment groups (**Figure 5E**), suggesting a polarization shift toward a more pro-inflammatory, anti-tumor macrophage phenotype. These results indicate that, in the EO771 TNBC *in vivo* model, stemness inhibitors may favorably reprogram the macrophage population.

We also observed interesting dynamics in the dendritic cell population. Both JIB-04 and SB-431542 treatments significantly elevated the relative proportion of total intratumoral dendritic cells compared to other treatment groups (p<0.05), although the effect of SB-431542 was less pronounced (**Figure 5H**). Comprehensive analysis of dendritic cell subpopulations revealed that both SB-431542 and JIB-04 treatments increased the proportion of conventional DC1 (cDC1) cells (**Figure 5I**), which are critical for antigen presentation and activation of anti-tumor immunity. However, this increase was not observed in the quadruple combination group, where cDC1 levels were comparatively lower (p<0.05). Other dendritic cell populations, such as cDC2 (**Figure 5J**), did not exhibit significant changes, but JIB-04 treatment notably increased the levels of monocyte-derived dendritic cells (p<0.05, **Figure 5K**) and plasmacytoid dendritic cells (p<0.05, **Figure 5L**) which could contribute to immune regulation within the tumor. No significant differences were found in neutrophil (**Figure 5F**) or monocyte (**Figure 5G**) populations across treatment groups, indicating that the effects of the stemness inhibitors were more pronounced on macrophages and dendritic cells.

Overall, the findings from this *in vivo* study align with our hypothesis that stemness inhibitors could modulate the immune microenvironment, particularly by reprogramming macrophage polarization. The increased M1/M2 ratio in the SB-431542 and combination groups suggests that treatments based on TGF-β blockade may help to shift the balance toward a more anti-tumor immune response. Furthermore, the increase in cDC1 cells with SB-431542 and JIB-04 treatments indicates their potential for enhanced antigen presentation and immune activation. This highlights the potential of stemness inhibitors, particularly when those targeting different stemness pathways are combined, to reprogram the TME as part of a broader, more comprehensive therapeutic strategy.

## 4 Discussion

This study uniquely explores how cancer cell lines influence macrophage polarization within the TME, focusing on the immune-modulatory properties of tumor cells. Macrophages play a central role in shaping the immune response in cancer, often adopting a spectrum of phenotypes influenced by the surrounding environment (3). Heavy infiltration with immunosuppressive macrophages is related with poor prognosis in most frequently diagnosed cancers, such as colorectal (48) and breast (49). Our investigation into the macrophage polarization potential of CRC cells *in vitro* demonstrated that cell lines representing the mesenchymal CMS4 subtype and expressing the CD44 stemness marker strongly promote M2-like macrophage polarization, consistent with phenotypes observed in TCGA patient cohort. Furthermore, this research stands as one of the initial investigations to describe how specific stemness inhibitors not only reduce cancer cell stemness but also modulate macrophage polarization, offering important clinical implications. By reprogramming macrophages from an M2-like to an M1-like phenotype in CRC cells *in vitro* and in a breast tumor model *in vivo*, these inhibitors could potentially reshape the TME to favor anti-tumor immunity and enhance the effectiveness of existing immunotherapies.

Our findings suggest that stemness properties in CRC cells influence macrophage polarization and may contribute to a more complex immune landscape in CMS4 tumors. Importantly, stemness-driven macrophage polarization toward the M2-like phenotype has also been observed in other cancer types, further supporting its significance across different tumor localizations: ovarian (50–52), cervical (53), hepatocellular (54), lung (55), and breast (56). Although we did not provide the direct mechanistic explanation for this phenomenon, other studies suggest showed that cancer stem cells secrete cytokines such as IL-6 and IL-10, which activate key signaling pathways in macrophages, particularly STAT3 and NF-κB (57, 58) and in turn drives the polarization of M2 macrophages (18). NF-κB signaling is triggered in macrophages by tumor-derived cytokines such as TNF-α and IL-1β, further enhancing the production of IL-6 and IL-10, that further promote tissue remodeling and angiogenesis (18, 56, 59). This creates a self-sustaining interplay between stemness-associated signaling in cancer cells and M2 macrophage polarization *in vivo*. In our transwell co-culture experiment, however, we did not note the association between elevated IL-6 or IL-10 production in CRC cells and their macrophage polarization potential. Interestingly, the HCT116 cell line, which exhibited the strongest ability to induce M2-like macrophage polarization, produced high levels of IL-4 and IL-13 –cytokines known to drive M2-like polarization in macrophages via the STAT6-dependent JAK/STAT signaling pathway (60).

HCT116 and SW620 cell lines are classified as representatives of the mesenchymal CMS4 subtype of colorectal cancer (37) However, they exhibited notable differences in surface marker expression and immunomodulatory properties when co-cultured with macrophages. HCT116 cells were predominantly CD44+ and secreted high levels of IL-4, IL-13, CCL8, CCL13, AXL, and EGF, which promoted strong M2-like macrophage polarization in transwell co-culture. In contrast, SW620 cells, which were mostly CD44-, expressed proinflammatory mediators such as TNF and CXCL9, favoring M1-like polarization. These differences may be attributed to unique genetic mutations and active signaling pathways in these cell lines. HCT116 cells harbor mutations in KRAS, PIK3CA, TGFBR2, and CTNNB1, activating the PI3K/AKT, Wnt/β-catenin, and TGF-β pathways, which promote cell survival, proliferation, and immune modulation (61, 62). In contrast, SW620 cells, derived from a metastatic site, carry mutations in KRAS, APC, and TP53, activating the RAS/RAF/MEK/ERK and Wnt/β-catenin pathways, leading to cell proliferation, genomic instability, and metastasis (62, 63). Similar findings of CRC cells differentially influencing macrophage polarization have been reported in other *in vitro* and *in vivo* studies (12, 64–66), highlighting the and intricate interplay between cancer cells and the TME.

Monotherapy and combination therapy using stemness inhibitors revealed several significant effects on CRC cell lines. We found that the quadruple combination of salinomycin, SB-431542, JIB-04, and napabucasin most effectively reduced stemness markers such as CD133 and CD44. Notably, despite single inhibitors have been reported effective at reducing surface stemness markers (67), previous studies have highlighted the enhanced efficacy of combination therapy over monotherapy in targeting cancer stemness pathways (38, 42, 68). Our data suggest that stemness inhibition affects not only stemness markers expression on cancer cells but also modulates immune evasion strategies via MHC I regulation. MHC I molecules are crucial for presenting tumor antigens to cytotoxic T cells, and their downregulation is a common mechanism employed by tumors to escape immune detection (69). In our study, we observed reduced MHC I expression in HCT116 cells, which may contribute to immune evasion, while in SW620, MHC I expression increased, potentially enhancing immune surveillance. These divergent effects on MHC I further emphasize the distinct response between HCT116 and SW620 and indicate that stemness inhibitors could differentially modulate immune recognition in CMS4 tumors, a subtype often characterized by immunosuppressive TME and poor response to immunotherapy (12).

Current literature lacks precise insights into the effects of stemness inhibitors on macrophages. Shen et al. demonstrated that salinomycin, at low doses, could reprogram tumor-associated macrophages toward an M1-like phenotype (70). Similarly, in our study, we showed that a combination of stemness inhibitors could induce macrophage polarization and shift the tumor microenvironment toward a more immune-responsive state. Our study is among the first to demonstrate the impact of combining multiple stemness inhibitors, including salinomycin, SB-431542, JIB-04, and napabucasin, on modulating immune polarization in CMS4 CRC subtype. Specifically, the quadruple combination showed the strongest reprogramming effects, reducing M2 markers (CD206, CD163) and increasing M1 markers (CD86, CD11c, HLA-DR) in the indirect co-culture with HCT116 cells, suggesting a shift toward anti-tumor immunity. This supports the hypothesis that targeting stemness pathways can influence the immune TME by altering macrophage polarization, a finding that could inform future therapeutic strategies for CMS4 tumors.

To observe whether the macrophage polarization and stemness inhibition phenomena *in vitro* can be reflected in a more complex biological system, we extended our study to an *in vivo* setting. We used the EO771 triple-negative breast cancer mouse model to assess the broader effects of stemness inhibition on the immune landscape of the tumor microenvironment. While acknowledging that this may not reflect colorectal cancer TME dynamics, EO771 was selected for its immunocompetent, macrophage-rich environment and high CD44 expression, avoiding the limitations of immunocompromised xenograft models. The most notable observation upon treating tumor-bearing mice with stemness inhibitors was the considerable reduction of intratumoral M2 macrophages resulting in substantially increased M1/M2 macrophage ratio in groups treated with SB-431542 and quadruple combination. This shift underscores the capacity of stemness inhibitors to favorably modulate the tumor microenvironment. Although SB-431542’s potential to inhibit tumor growth has been demonstrated previously, its effects were primarily attributed to modulating regulatory T and B cells (71) and promoting dendritic cell maturation (72), rather than acting directly on macrophages.

Additionally, we observed a considerable increase in conventional DCs type 1 (cDC1) following treatment with SB-431542 and JIB-04. cDC1 play a critical role in cross-presenting tumor antigens and priming CD8+ T cells for anti-tumor activity (73). The recruitment and activation of cDC1 by stemness inhibitors may enhance the cytotoxic T cell response, creating a positive feedback loop that sustains anti-tumor immunity. Interestingly, the relative proportion of cDC1 diminished in the quadruple combination group, suggesting potential interactions between these inhibitors that may affect dendritic cell dynamics differently. Moreover, JIB-04 not only significantly increased cDC1 levels but also promoted the expansion of other dendritic cell subsets, such as conventional DCs type2, monocytic DCs, and plasmacytoid DCs, which can have context-dependent roles in modulating the antitumor immune response (74). However, their precise contributions within the TME require further elucidation, particularly for plasmacytoid DCs, which are known to exhibit tolerogenic properties in certain scenarios (75). Overall, while stemness inhibitors did not affect neutrophil or monocyte populations, SB-431542 consistently reduced M2 macrophages and increased cDC1 proportion, whereas JIB-04 selectively expanded DC subsets. These findings suggest that stemness inhibitors can specifically modulate distinct myeloid cell populations within the tumor microenvironment.

The observed reprogramming of macrophages toward an M1-like phenotype upon treatment with stemness inhibitors *in vitro* and *in vivo* may have broader implications for immune modulation within the TME. By shifting the balance of macrophages, these inhibitors could indirectly influence other immune cell populations, particularly T cells and neutrophils. Cancer stem cells were shown to contribute to T cell dysfunction by promoting IL-10 and TGF-β (76), which inhibit CD8+ cytotoxic T cells and induce the expansion of Tregs. However M1 macrophages are known to produce cytokines such as IL-12, TNF-α, and IFN-γ, which not only suppress Treg expansion but also impair their immunosuppressive function (77). This cytokine-mediated disruption of Tregs reduces their production of IL-10, thereby alleviating CD8+ T cell suppression and promoting their activation and cytotoxic function. As a result, M1 macrophages play a crucial role in altering the Treg/CD8+ cell ratio in the tumor microenvironment (78). Such a shift in the Treg/CD8+ ratio is critical for enhancing anti-tumor immune responses, particularly in CMS4 CRC tumors where T cell dysfunction is a hallmark of the immunosuppressive microenvironment (12, 79). Additionally, M1 macrophages can recruit and activate neutrophils, driving their polarization toward an N1-like phenotype characterized by anti-tumor activity (80). N1 neutrophils secrete ROS, TNF-α, and other cytotoxic molecules that contribute to tumor cell killing (81). Taken together, by reprogramming macrophages from an M2-like to an M1-like phenotype, stemness inhibitors used as a complementary strategy have the potential to overcome TME-mediated barriers and enhance anti-tumor immune responses, particularly in immunosuppressed and immunotherapy-resistant cases such as CMS4 CRC (82, 83) or other mesenchymal-like tumors.

Although this study provides insights into macrophage polarization and stemness inhibition, several limitations need to be acknowledged, such as the use of an indirect co-culture system limits cell-to-cell contact, underrepresenting the complexity of the TME. Moreover, the limited range of selected models (two CRC cell lines and one breast cancer model) restricts the generalizability of the findings. Further research using a broader panel of cell lines, as well as other cancer types, would be essential to confirm whether the observed effects on macrophage polarization and stemness inhibition extend beyond these specific models. While the EO771 breast cancer model facilitated exploration in an immunocompetent setting, it did not fully reflect the unique dynamics of the CRC TME, particularly the highly stromal and immunosuppressive features of CMS4 tumors. Future studies using CRC-specific *in vivo* models, such as syngeneic murine CRC models or orthotopic organoid-derived systems, would better mimic these interactions and provide more clinically relevant insights.

Furthermore, our results provided the initial insights into the hypothesis of stemness-related macrophage M2 polarization and its reprogramming, but additional studies are needed to fully explore the mechanistic basis and broader implications of stemness inhibition in the TME. While changes in macrophage surface markers were assessed, no functional assays were conducted to validate whether these phenotypic shifts translate into improved anti-tumor activity. Finally, though macrophages were the primary immune cell type studied, other key immune cells within the TME, such as T cells, were not examined, which could limit the broader implications of the findings for stemness inhibition-induced immune modulation.

In conclusion, our findings demonstrate that stemness inhibitors not only target cancer cells but also modulate the immune microenvironment by reprogramming macrophages. This dual effect highlights the therapeutic potential of stemness-targeting strategies, particularly in managing the CMS4 (mesenchymal-like) CRC tumors, where manipulating TME emerges as the promising field of exploration (82). The ability of stemness inhibitors, especially SB-431542 and salinomycin, to shift macrophage polarization from an M2-like to an M1-like phenotype could synergize with existing immunotherapies, such as checkpoint inhibitors, represents an exciting area of future exploration. Moreover, expanding these findings to other colorectal cancer subtypes and additional tumor models will help determine whether these effects are specific to CMS4 or have broader applicability. Investigating the molecular mechanisms underlying these immune-modulatory effects, particularly the signaling pathways involved, will be essential for refining these therapies and optimizing their clinical use.

## Data Availability

The data presented in the study are deposited in the ArrayExpress repository, accession number E-MTAB-14915.
