# Supplementary material for: Targeting stemness pathways modulates macrophage polarization and reprograms the tumor microenvironment
Source: Front Immunol. 2025 Mar 14;16:1513404. doi: 10.3389/fimmu.2025.1513404 (PMC11950675; doi:10.3389/fimmu.2025.1513404)
Supplement: Supplementary file 1 [file DataSheet1.docx]

Supplementary Material

# Supplementary Figures


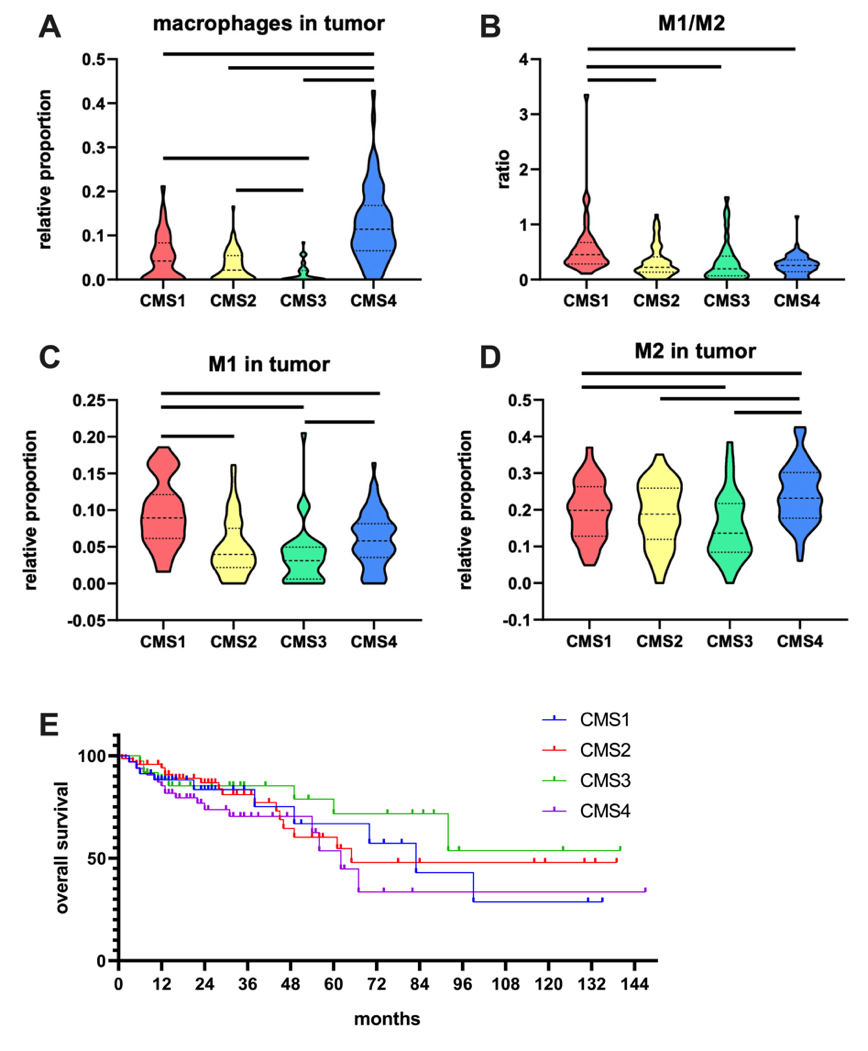


**Supplementary Figure 1.** **Macrophage landscape and survival analysis in TCGA colorectal cancer cohort.** Violin plots illustrating relative proportions of macrophages, deconvoluted using TIMER2.0, across annotated colorectal cancer Consensus Molecular Subtypes (CMS1 n=76, CMS2 n=220, CMS3 n=72, CMS4 n=144) in TCGA-COAD tumor samples (A-D). (A) The overall proportion of macrophages is highest in CMS4 tumors compared to other subtypes. (B) The M1/M2 ratio was the highest in CMS1 tumors, with CMS4 tumors significantly lower, indicative of immune-suppressive environments. (C) CMS1 tumors have the highest relative proportion of M1 macrophages, followed by CMS4, while CMS3 has the lowest. (D) CMS4 tumors exhibit the highest proportion of M2 macrophages, indicating a predominantly immune-suppressive intratumoral macrophage landscape. Statistical significance was tested using one-way ANOVA with post-hoc analysis and is denoted by horizontal lines across the subtypes (p < 0.05). (E) The Kaplan-Meier plot illustrates the overall survival of the TCGA cohort stratified into CMS1-4 groups over time (months), log-rank test did not indicate differences in survival (p = 0.301).

**
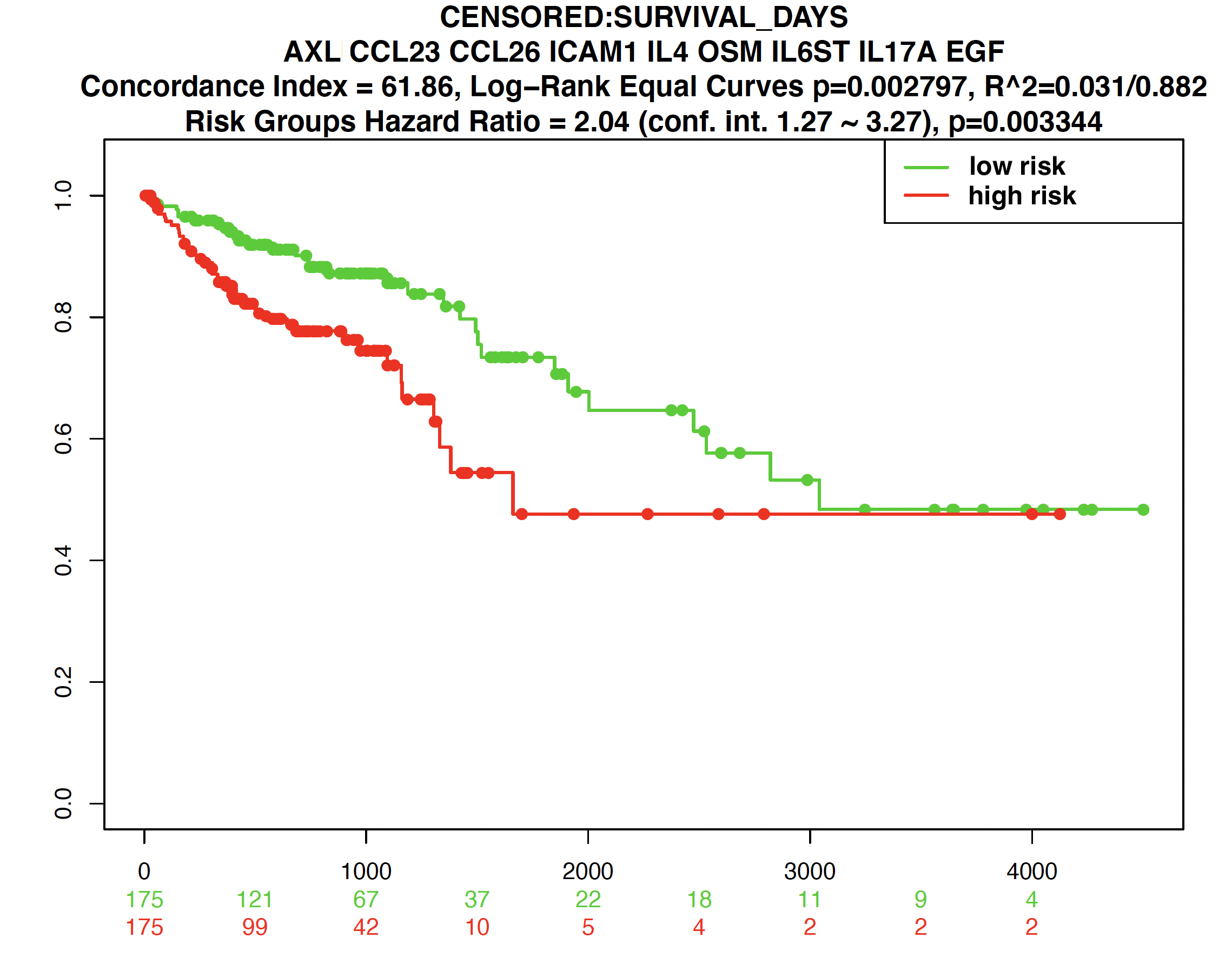
**

**Supplementary Figure 2.** **Prognostic value of genes encoding for derived macrophage polarization-related cytokine signature.** SurvExpress tool was used to correlate the gene expression of a selected cytokine panel with overall survival in TCGA colorectal cancer patients. The cohort is stratified into high- and low-risk groups based on cytokine-encoding gene expression levels. The Kaplan-Meier plot illustrates the survival distribution between these groups over time (days), with the log-rank test indicating significant differences in survival (p < 0.05). A concordance index of 61.86 was calculated, suggesting moderate accuracy in survival prediction.


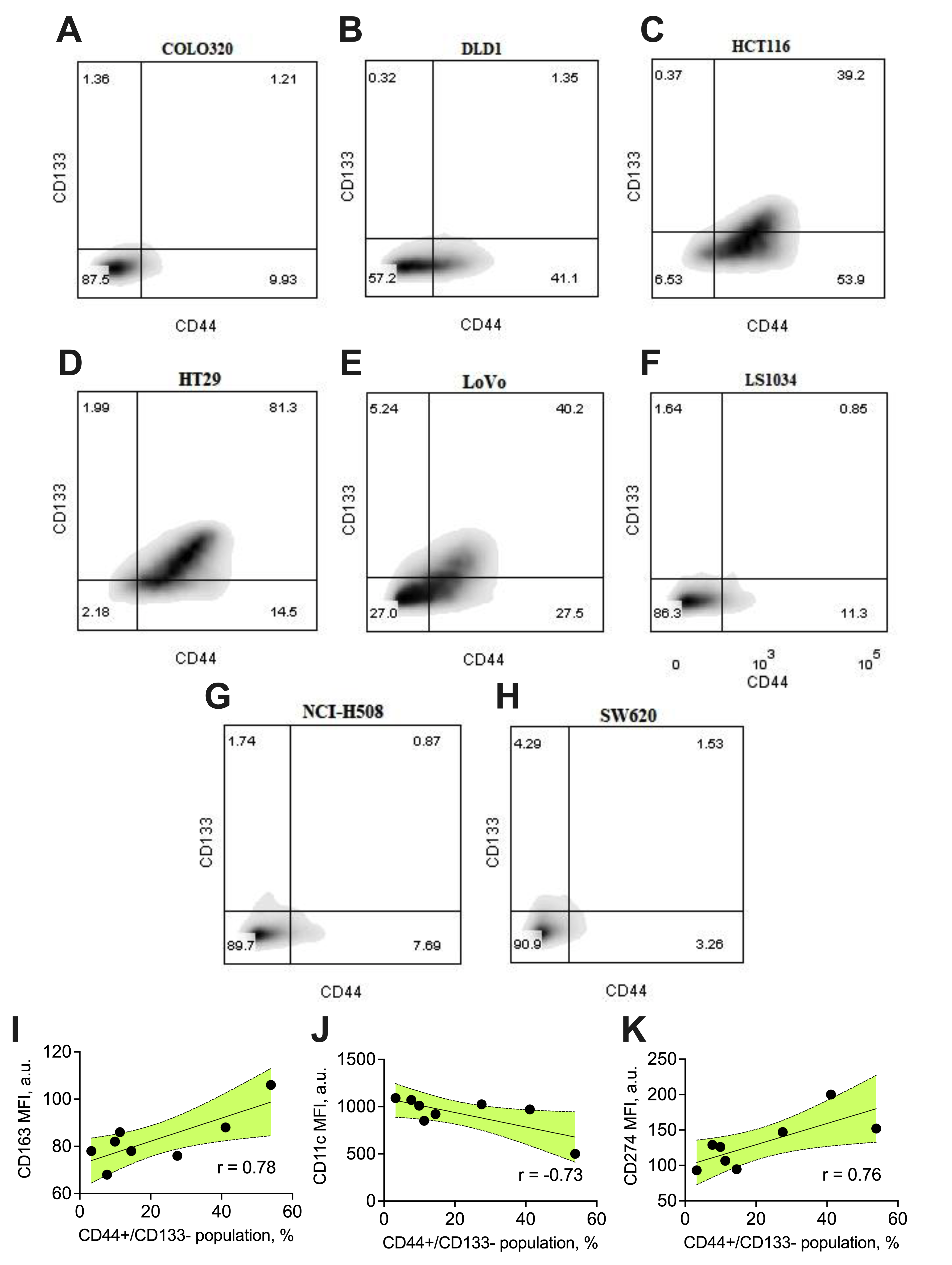


**Supplementary Figure 3.** **Colorectal cancer cell lines stemness-associated phenotype and correlation with macrophage marker expression.** Flow cytometry plots showing the expression of CD44 (X axis – fluorescence intensity, a.u) and CD133 (Y axis – fluorescence intensity, a.u) in COLO320 (A), DLD1 (B), HCT116 (C), HT29 (D), LOVO (E), LS1034 (F), NCI-H508 (G), SW630 (H) cell lines. The quadrants display the percentage of CD133 and CD44 positive cells in each. (B) Correlation analysis between the percentage of CD44+/CD133- CRC cells (X-axis) and macrophage marker CD163 (I), CD11c (J), CD274 (K) expression (Y-axis, MFI values). Pearson correlation coefficients (r) are displayed in each plot, with shaded areas representing the 95% confidence interval. All shown correlations were statistically significant (two-sided p < 0.05, Pearson correlation test), indicating strong positive or negative associations between the size of the CD44+/ CD133- population and specific macrophage polarization markers. A.u. – arbitrary units, CRC – colorectal cancer, MFI – median fluorescence intensity.


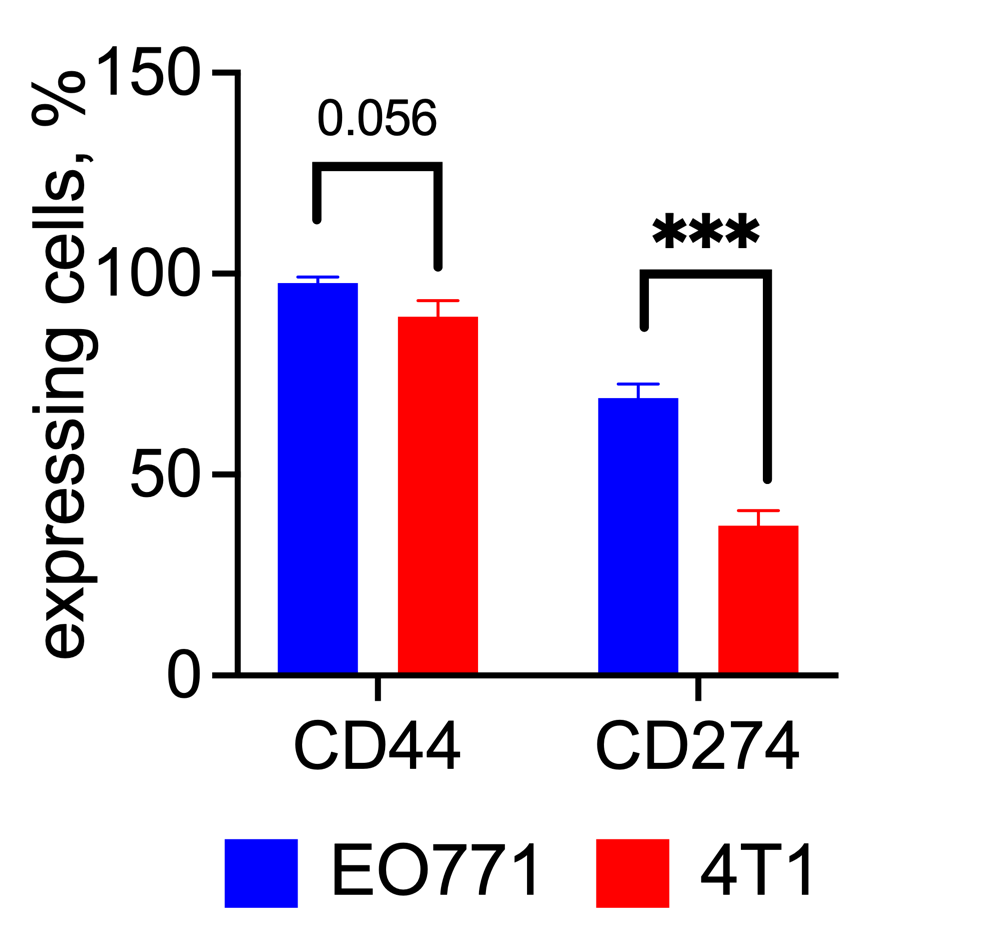


**Supplementary Figure 4.** **Phenotype of mouse breast cancer cell lines.** Flow cytometric analysis of EO771 and 4T1 murine breast cancer cells showing the expression of CD274 and CD44 markers. Each bar represents the median percentage of marker-positive cells with error bars indicating interquartile range from three independent replicates. Statistical significance is denoted (**p < 0.01).
